# Community-Based Evaluation of PMTCT Uptake in Nyanza Province, Kenya

**DOI:** 10.1371/journal.pone.0110110

**Published:** 2014-10-31

**Authors:** Pamela K. Kohler, John Okanda, John Kinuthia, Lisa A. Mills, George Olilo, Frank Odhiambo, Kayla F. Laserson, Brenda Zierler, Joachim Voss, Grace John-Stewart

**Affiliations:** 1 Global Health and Psychosocial & Community Health, University of Washington, Seattle, Washington, United States of America; 2 Center for Global Health Research, Kenya Medical Research Institute, Kisumu, Kenya; 3 Kenyatta National Hospital/University of Nairobi, Nairobi, Kenya; 4 Kenya Medical Research Institute/Centers for Disease Control and Prevention (KEMRI/CDC) Research and Public Health Collaboration, Kisumu, Kenya; and Division of HIV/AIDS Prevention, CDC, Atlanta, Georgia, United States of America; 5 Biobehavioral Nursing & Health Systems, University of Washington, Seattle, Washington, United States of America; 6 Global Health, Medicine, Pediatrics, and Epidemiology, University of Washington, Seattle, Washington, United States of America; UCL Institute of Child Health, University College London, United Kingdom

## Abstract

**Introduction:**

Facility-based assessments of prevention of mother-to-child HIV transmission (PMTCT) programs may overestimate population coverage. There are few community-based studies that evaluate PMTCT coverage and uptake.

**Methods:**

During 2011, a cross-sectional community survey among women who gave birth in the prior year was performed using the KEMRI-CDC Health and Demographic Surveillance System in Western Kenya. A random sample (n = 405) and a sample of women known to be HIV-positive through previous home-based testing (n = 247) were enrolled. Rates and correlates of uptake of antenatal care (ANC), HIV-testing, and antiretrovirals (ARVs) were determined.

**Results:**

Among 405 women in the random sample, 379 (94%) reported accessing ANC, most of whom (87%) were HIV tested. Uptake of HIV testing was associated with employment, higher socioeconomic status, and partner HIV testing. Among 247 known HIV-positive women, 173 (70%) self-disclosed their HIV status. Among 216 self-reported HIV-positive women (including 43 from the random sample), 82% took PMTCT ARVs, with 54% completing the full antenatal, peripartum, and postpartum course. Maternal ARV use was associated with more ANC visits and having an HIV tested partner. ARV use during delivery was lowest (62%) and associated with facility delivery. Eighty percent of HIV infected women reported having their infant HIV tested, 11% of whom reported their child was HIV infected, 76% uninfected, 6% declined to say, 7% did not recall; 79% of infected children were reportedly receiving HIV care and treatment.

**Conclusions:**

Community-based assessments provide data that complements clinic-based PMTCT evaluations. In this survey, antenatal HIV test uptake was high; most HIV infected women received ARVs, though many women did not self-disclose HIV status to field team. Community-driven strategies that encourage early ANC, partner involvement, and skilled delivery, and provide PMTCT education, may facilitate further reductions in vertical transmission.

## Introduction

Global elimination of mother-to-child HIV transmission (MTCT) is targeted for 2015 and is an initiative which will require strategic improvements in service delivery. [1] As PMTCT interventions have expanded globally, challenges in delivery and uptake of services have persisted. The World Health Organization (WHO) estimated that in 2010, in low and middle income countries, only 35% of pregnant women received HIV testing, and less than half of HIV infected women tested accessed antiretrovirals (ARVs) for PMTCT. [2] By 2012, the 21 Global Plan for Elimination of Pediatric HIV priority countries reported 64% coverage, however the pace of decline in the number of newly infected children has been slow in some countries. [3] To meet elimination (eMTCT) goals, a focus in sub-Saharan Africa is critical given the high HIV prevalence among women of childbearing age in this region.

PMTCT programs involve a cascade of interventions, which begins with HIV counseling and testing of pregnant women at initiation of antenatal care (ANC), and provision of ARVs throughout pregnancy, peripartum, and in the postpartum period to prevent vertical HIV transmission. [4] Modeling suggests that improving PMTCT coverage throughout the cascade would decrease infant HIV more than improving maternal antiretroviral regimens. [5] The Kenya Ministry of Health has made great strides to increase PMTCT coverage, with provision of ARVs to HIV-infected pregnant women increasing from 20% uptake in 2005 [6] to 69% in 2011. [7] As a result, new child HIV infections in Kenya have decreased, averting 46,000 new infections since the introduction of PMTCT. [7] However, the number of newly infected children per year remain high (13,000 in 2012), [3] and recent estimates of MTCT rates, while ≤5% in clinical trial settings, [8]–[11] range from 8 - 27% in Kenyan surveys [7], [12]–[14].

Most PMTCT assessments are clinic-based, and few studies sample a broader population of mothers which includes those who never accessed clinics. While clinic-based assessment is informative, this can exclude vulnerable and underserved women, overestimating PMTCT coverage and failing to adequately capture barriers to accessing PMTCT. A study from Uganda noted markedly lower estimates of antenatal HIV testing in community-based assessment than in clinic-based assessment. [15] Another recent study in Cameroon, Cote D'Ivoire, South Africa, and Zambia similarly found that facility-based estimates of PMTCT coverage exceeded coverage estimates observed in the community [16].

Expansion of PMTCT coverage in Kenya faces persistent barriers due to social, cultural, programmatic, logistical, and policy challenges also seen in other sub-Saharan countries. [17]–[23] To complement a clinic-based assessment conducted in 2009, [24] this survey aimed to assess PMTCT coverage and barriers to access to PMTCT from a community perspective.

## Methods

### Study Design

A cross-sectional community-level survey assessing knowledge and uptake of PMTCT services among women of child-bearing age was performed March – June, 2011 in the Kenya Medical Research Institute (KEMRI) and US Centers for Disease Control (CDC) Health and Demographic Surveillance System (HDSS) area in Nyanza Province, Kenya.

### Study Location

The HDSS covers 385 villages with a population of approximately 220,000. Three regions situated in Siaya County make up this area: Karemo, Gem and Asembo. [25] All three regions are rural. The HDSS was launched in September 2001 by the US Centers for Disease Control and Prevention (CDC) in collaboration with the Kenya Medical Research Institute (KEMRI) and serves as a community-based platform. The HDSS provides demographic and health information as well as disease- or intervention-specific information. Since its inception, the HDSS has collected 13 years of population-level data. Home-based HIV counseling and testing (HBCT) of the HDSS population has been conducted since 2008, but had not been implemented in all areas at the time of the study. The HDSS area includes 41 health facilities that provide care to pregnant women.

### Study Population

Women, maternal age 14 years and older (mothers 14–17 are considered emancipated minors), who were residents in the HDSS area, and had delivered a baby within the previous year (January to December 2010) were recruited. Residency was defined as having lived in the area for at least 4 consecutive months. We leveraged existing HDSS program data to identify and recruit two groups representing two target populations for prevention services: a random community sample to assess factors influencing uptake of general services (access to ANC and maternal HIV testing); and a second sample of HIV infected women, known via previous home-based testing and counseling in the region, to assess factors influencing uptake of HIV-specific services (use of ARVs and infant HIV testing). The same inclusion criteria applied to both general community and HIV-positive groups, with the addition that women in the HIV-positive group must have completed HIV-testing prior to the delivery of the infant.

### Sampling

Sampling was limited by the number of women meeting recruitment criteria in the HDSS database. We were able to generate a random list of 523 women from non-HBCT areas to represent a general community assessment of ANC and HIV-testing uptake. In HBCT areas, only 275 women were HIV positive, thus all HIV positive mothers who were diagnosed prior to delivery were approached.

### Data Collection

Trained fieldworkers were assigned a list of names and locations for all selected participants. Village reporters assisted fieldworkers to locate the mother participant and introduce the study. Fieldworkers administered surveys and recorded GPS location on hand-held PDAs using electronic forms (Pendragon Software Corporation, Buffalo Grove, IL); paper forms were used as a back-up. Mothers' surveys were adapted from clinic-based surveys used previously in this region [24]. Women were surveyed regarding their knowledge, opinions and use of ANC and PMTCT services at last pregnancy.

Outcomes of interest included self-report of uptake of ANC, HIV testing, maternal ARVs for PMTCT, infant testing, and infant ARVs. Potential cofactors assessed included demographics, educational achievement, and marital status, as well as knowledge about HIV and disease transmission. Due to limitations in determining income through household surveys, asset-based indicators (ownership of goods, including mobile phones, cattle, television or refrigerator, and roof type) were used as measures of socioeconomic status. [26], [27] Fieldworkers were not informed of HIV status of participants, thus participant self-reported HIV status was used. In 2010, Kenya national PMTCT guidelines adopted WHO PMTCT Option A (zidovudine during pregnancy with infant nevirapine during breastfeeding for women without advanced HIV, or triple-drug antiretroviral therapy for women with advanced disease) with a provision to implement Option B (triple-drug antiretroviral therapy for all women, stopping after breastfeeding for those without advanced disease) in higher resource systems. As national adoption of new guidelines was slow, and only half of national facilities offered PMTCT, [12] we chose to assess self-report of any maternal ARV uptake at each of three time-points: antenatal, perinatal, and postpartum.

### Data Analysis

Descriptive proportions of those accessing services in the PMTCT cascade were generated. Data analysis utilized chi-square analyses with Fisher's exact tests for comparison of proportions using STATA SE version 11 (STATACorp, College Station, Texas). Wilcoxon Mann-Whitney tests were used for comparisons where continuous data were not normally distributed. Multivariate analysis using generalized linear models further assessed adjusted prevalence ratios of uptake. A priori covariates of age and education level were included in the model with correlates identified in univariate analyses. Variables were retained in the model if they were significantly associated with the outcome and/or if their inclusion substantially changed the estimates by 10%.

Analysis was modeled to assess key steps in the PMTCT cascade: correlates of ANC attendance were assessed among women in the community sample (n = 405); correlates of HIV testing were assessed among women attending ANC with unknown or previously negative HIV status (n = 362); and correlates of maternal and infant ARV uptake were assessed among self-identified HIV-positive women (n = 216).

### Ethical Considerations

Prior to study start, community engagement activities were held targeting the area senior health officials, the local community advisory board, the village reporters, chiefs and assistant chiefs. Written informed consent was obtained from all study participants, both to be interviewed and also to have their data from these surveys linked to their HDSS record. In Kenya, women with pregnancy are considered emancipated and were therefore able to consent to study participation without parental assent. All study procedures, including enrollment of emancipated minors, were approved by the University of Washington Institutional Review Board (#36022) and the Kenya Medical Research Institute Ethical Review Committee (#1714). Written permission was also received from provincial medical and public health offices.

## Results

### Enrollment

**Figure 1:**
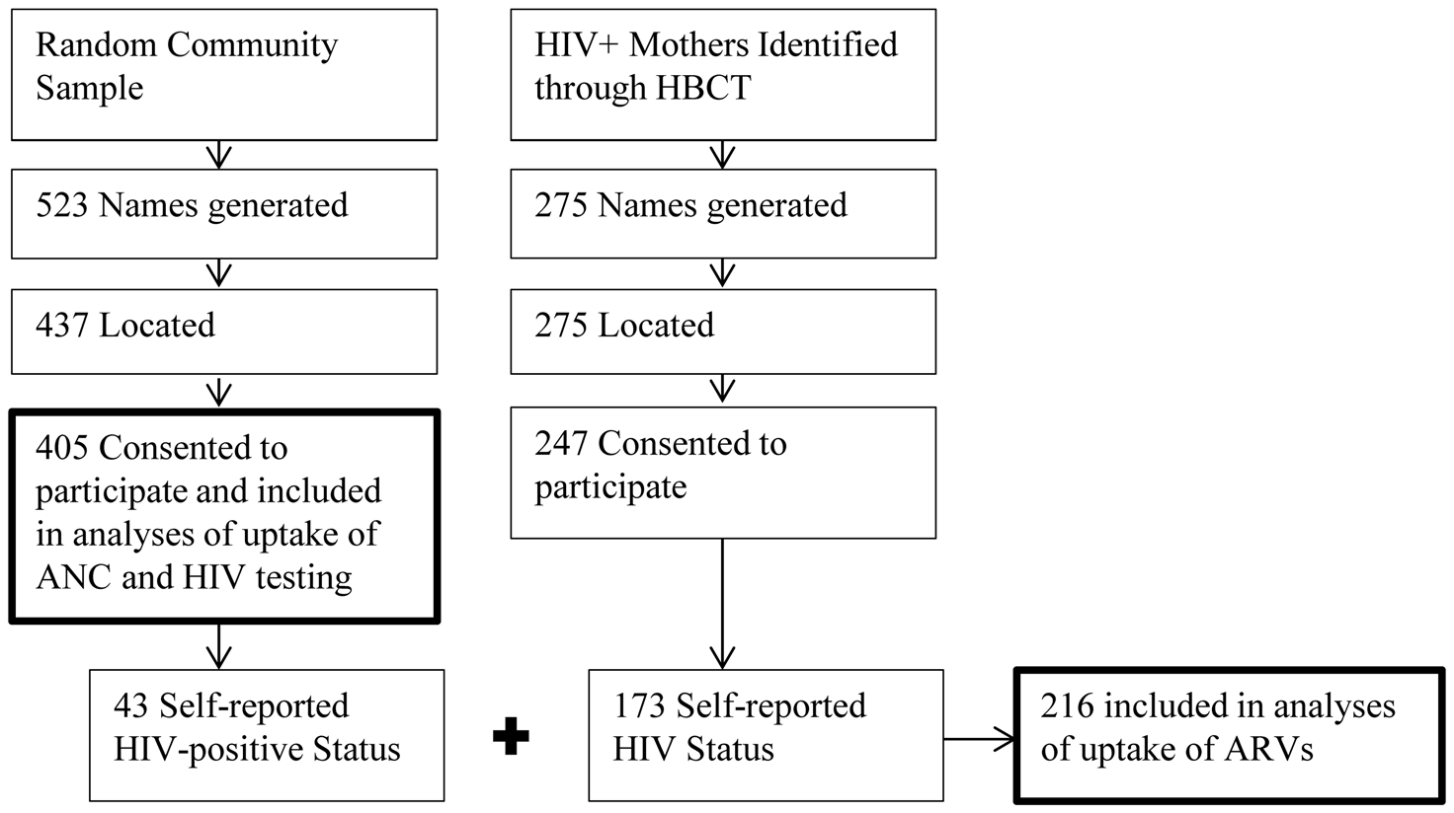
Sampling and Enrollment.

A random sample of 523 women who delivered in the previous year was identified, 437 (83.6%) were located, and 405 (92.7% of those located) agreed to participate (Figure 1). Among 275 women known to be HIV infected through prior HBCT, 247 (89.8%) consented to participate. However, only 173 (70%) of those enrolled reported to fieldworkers that they were HIV-positive and subsequently answered questions about PMTCT. Thus, the HIV-infected sample includes 43 women from the random community sample and 173 women from the HBCT sample to total 216 women.

### Population Characteristics

Among the 405 women in the general community sample, most were married (79% monogamous and 15% polygamous) (Table 1). The majority were of poor socioeconomic status; 42% held any job or small-business (less than 2% were salaried workers), and less than half of the women had completed a primary education. The median number of mobile phones per household was 1 (IQR 0-2), with 71% of women reporting one or more phones in the household. Under half of households reported cattle ownership (median 0, IQR 0-2), and >90% of households did not own a television or refrigerator. Approximately one third (29%) had better than a grass roof (a marker of higher socioeconomic status), and all but one of these were iron sheet.

**Table 1:** Population and Sample.

*Participant Characteristic*	*Community Sample*	*HIV+ Mothers* *
	*(n = 405)*	*(n = 216)*
	n(%) or median (IQR)	n(%) or median (IQR)
**Demographic Information**		
Age	25 (22–30)	29 (25–32)
Years of residence in village	7.2 (3.5–12)	9.5 (5–14)
Marital status		
Married monogamous	318 (78.5)	159 (73.6)
Married polygamous	60 (14.8)	35 (16.2)
Single	17 (4.2)	7 (3.2)
Widowed	10 (2.5)	15 (6.9)
Occupation		
Unemployed	71 (17.5)	39 (18.1)
Housewife	163 (40.3)	98 (45.4)
Salaried job or small business	170 (42.0)	77 (35.7)
Completed primary education	184 (45.4)	112 (51.9)
Cattle ownership (≥in household)	192 (47.4)	89 (41.2)
Mobile phone ownership (≥in household)	289 (71.4)	152 (70.4)
Roof type		
Grass	288 (71.1)	120 (55.6)
Corrugated iron sheet or better	116 (28.6)	96 (44.4)
**Pregnancy Information**		
Number of times pregnant	4 (3–6)	4 (3–6)
Any antenatal care last pregnancy	379 (93.6)	209 (96.8)
Number of ANC visits	3 (2–4)	3 (3–4)
Timing of first ANC visit (months pregnant)	5 (4–6)	5 (4–6)
Transport time to ANC (hours)	1 (0.5–1.5)	1 (0.5–1.5)
**HIV Testing**		
HIV tested last pregnancy	340 (84.0)	–
HIV test at first ANC visit (among those tested)	304 (89.4)	–
Tested at government facility (among those tested)	(86.2)	–
**Delivery Care**		
Delivery care		
Skilled attendant (nurse, doctor or midwife)	176 (43.5)	101 (46.8)
Unskilled attendant (family member or traditional birth attendant)	145 (35.8)	67 (31.0)
No assistance	45 (20.0)	45 (20.8)
Vaginal delivery (no instruments)	383 (95.3)	203 (94.0)
**Knowledge and Beliefs**		
Is there any way to prevent HIV (yes)	298 (73.6)	192 (88.9)
Can pregnant women give HIV to baby (yes)	261 (64.4)	172 (79.6)
Can ARVs prevent MTCT of HIV (yes)	210 (51.9)	158 (73.2)

*Includes 43 mothers from community sample and 173 mothers from HBTC.

Women who delivered an infant in the previous year and resided in the Demographic Health and Surveillance System Area, Nyanza Province, Kenya (2011).

Women reported a median of 4 (IQR 3-6) pregnancies. Although uptake of ANC was high (94%), most women started ANC late (median 5 months gestation at first ANC visit, IQR 4-6) and completed fewer than the recommended 4 ANC visits (median 3 visits, IQR 2-4). Uptake of skilled delivery, defined as a doctor, nurse or midwife, was 44%, with 20% reporting no assistance at all. Less than half of women reported delivering in a health facility (n = 170, 42.0%); 210 (52%) delivered at home and 14 (3.5%) delivered on the roadside while attempting to reach a health facility.

### PMTCT Cascade

**Figure 2:**
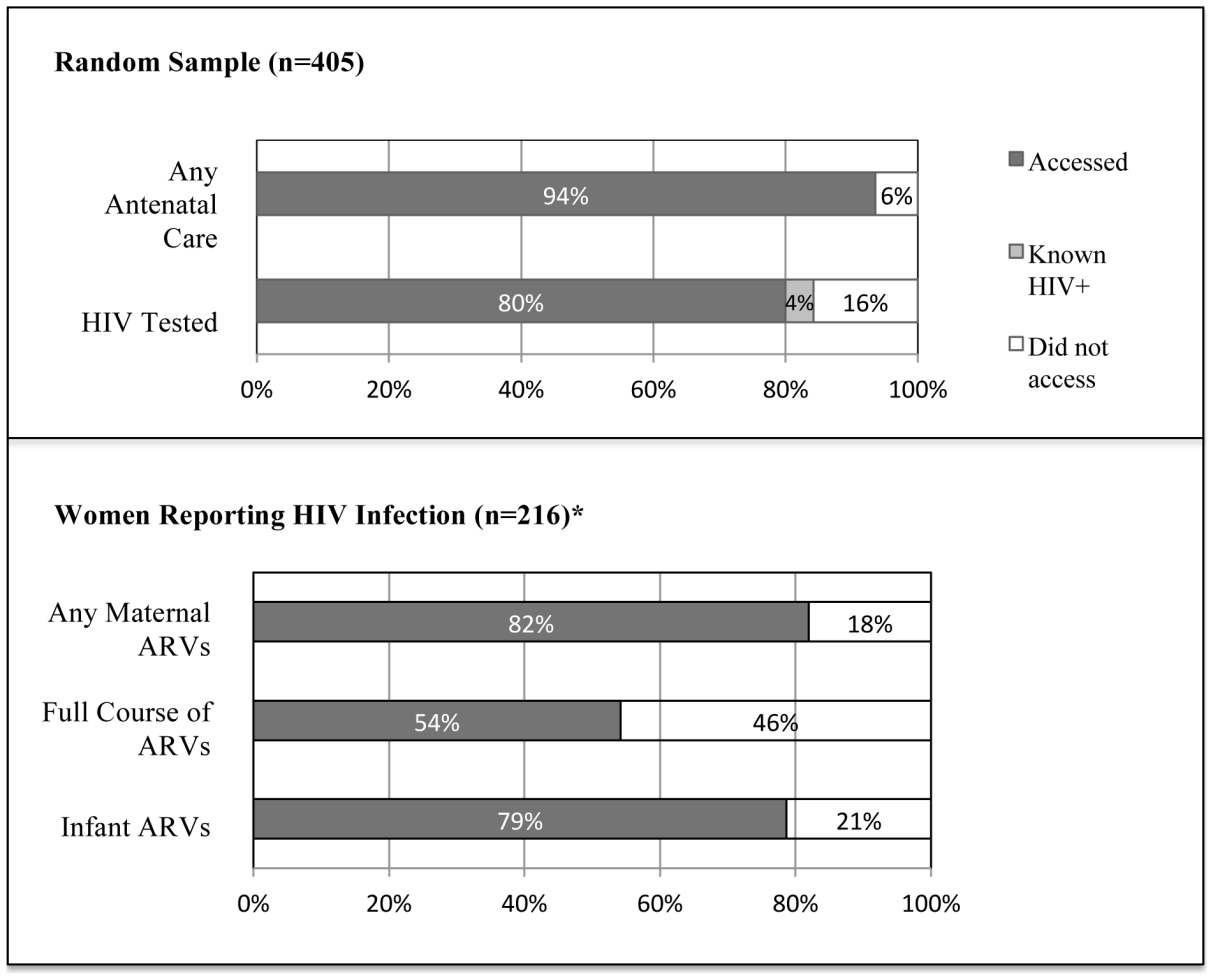
Uptake throughout cascade of PMTCT Services among Women in the Demographic Health Surveillance System Area, Nyanza Province, Kenya (2011). *Includes HIV+ oversample and does not reflect population HIV prevalence.

Most (94%) women from the random community sample reported attending ANC during the last pregnancy, among whom 89% reported that they were offered HIV testing and 87% reported being tested. Among women in the random sample, regardless of ANC attendance, 324 (80%) reported uptake of HIV testing, 4% were known positive, and 16% did not accept testing during the last pregnancy (Figure 2). Among women in the random sample who ever agreed to HIV testing, prevalence of HIV by self-report was 11% positive, 85% negative and 4% declined to answer.

Among 216 HIV-positive women, 82% reported receipt of maternal ARVs at any time-point (prenatal, labor and delivery, or postpartum), however uptake at all three time-points was considerably lower (54%). This was primarily driven by lower uptake during labor/delivery time-points (62% compared to 72% antenatal and 72% postpartum).

Seventy-nine percent of HIV-positive women reported administering infant ARVs for prevention of HIV transmission. Among the 81% (176/216) of infants tested for HIV, 11% of mothers reported that their infant was HIV-positive and 76% reported that the infant tested negative, 6% declined to report their child's HIV status, and 7% stated that they did not remember the test results. Timing of infant HIV diagnosis was not reported, though a national early infant diagnosis program with testing recommendations at six weeks was initiated in the previous year, December 2009. Among the 19 positive infants, 15 (79%) were reported to be receiving care and treatment for HIV, 1 child died prior to receiving treatment, and another 3 did not receive treatment because of fear of others finding out (n = 1), denial (n = 1), and being unaware of treatment (n = 2).

### Correlates of ANC Uptake in the Random Community Sample

Factors associated with report of ANC uptake at most recent pregnancy among women in the community sample are detailed in Table 2. In bivariate analyses, completion of primary education was associated with uptake of ANC. Among women who did not access ANC, 12% had completed primary education compared to 48% of women who accessed ANC (p<0.001) [Adjusted Prevalence Ratio (aPR):1.09; 95% Confidence Interval (CI): 1.04–1.15]. Women who did not access ANC reported a higher number of pregnancies than those who accessed ANC (median 5 vs. 4, p<0.001) (aPR: 0.94; 95%CI: 0.89–0.99).

**Table 2:** Reported uptake of antenatal care and HIV-testing in the random community sample.

*Population*	*Random community sample*			*Those who accessed ANC with negative or unknown HIV status*		
	*(n = 405)*			*(n = 362)*		
*Uptake of Service*	*No ANC*	*ANC*		*No HIV Test*	*HIV Tested*	
	*n(%) or med (IQR)*	*n(%) or med (IQR)*	*p-value*	*n(%) or med (IQR)*	*n(%) or med (IQR)*	*p-value*
	*26 (6.4)*	*379 (93.6)*		*48 (13.3)*	*314 (86.7)*	
**Demographic Information**						
Age	27 (23–32)	25 (22–30)	0.17	24.5 (22–28)	25 (22–30)	0.62
Years of residence in village	9 (4–13)	7 (3.5–11.3)	0.34	7 (3.5–10.3)	7 (3.3–11.5)	0.91
Marital status			0.63			0.23
Married monogamous	22 (84.6)	296 (78.1)		34 (70.8)	250 (79.6)	
Married polygamous	3 (11.5)	57 (15.0)		12 (25.0)	43 (13.7)	
Single	0 (0.0)	17 (4.5)		2 (4.2)	15 (4.8)	
Widowed	1 (3.9)	9 (2.4)		0 (0.0)	6 (1.9)	
Occupation			0.62			0.03
Unemployed	6 (23.1)	65 (17.2)		15 (31.3)	47 (15.0)	
Housewife	11 (42.3)	152 (40.2)		17 (35.4)	129 (41.2)	
Employed	9 (34.6)	161 (42.6)		16 (33.3)	137 (43.8)	
Completed primary education	3 (12.0)	181 (47.9)	<0.001	19 (39.6)	154 (49.2)	0.28
Cattle ownership (≥1 in household)	13 (50.0)	179 (47.5)	0.84	16 (33.3)	154 (49.4)	0.04
Mobile phone (≥1 in household)	15 (57.7)	274 (72.5)	0.12	24 (50.0)	236 (75.4)	<0.001
Roof type			1.00			0.06
Grass	19 (73.1)	269 (71.2)		40 (83.3)	217 (69.3)	
Corrugated iron sheet or better	7 (26.9)	109 (28.8)		8 (16.7)	96 (30.7)	
**Pregnancy Information**						
Number of times pregnant	5 (4–7)	4 (3–6)	0.001	3 (3–4.5)	4 (3–6)	0.23
**HIV Testing**						
Partner tested for HIV	–	–		16 (36.4)	179 (60.3)	0.003
Partner HIV positive (among tested)	–	–		1 (6.7)	19 (11.0)	1.00
**Knowledge and Beliefs** *						
Is there any way to prevent HIV	15 (60.0)	283 (75.1)	0.10	29 (61.7)	240 (76.7)	0.03
Pregnant women can give HIV to baby	14 (56.0)	247 (65.3)	0.39	28 (59.6)	205 (65.3)	0.51
Can prevent MTCT of HIV	10 (71.4)	200 (81.3)	0.48	16 (34.0)	171 (54.6)	0.01

* % women answering agree/yes to knowledge questions.

Women who delivered an infant in the previous year and reside in the Demographic Health and Surveillance System Area, Nyanza Province, Kenya (2011).

### Correlates of HIV Testing

Of 362 women attending ANC whose prior HIV status was negative or unknown, 87% reported being tested for HIV, and most HIV testing (89%) occurred at the first ANC visit (Table 2). Cofactors associated with HIV testing were occupation (31% versus 15% of those not accessing vs. accessing ANC, p = 0.03), mobile phone at home (50% vs. 75% of those not accessing vs. accessing ANC, p<0.001), partner having been tested for HIV (36% vs. 60% of those not accessing vs. accessing ANC, p = 0.003), and knowledge of PMTCT (34% vs. 55% of those not accessing vs. accessing ANC, p = 0.01). In adjusted models, socioeconomic indicators (cattle, phone ownership, and roof type) and report of partner HIV testing were significantly associated with an increased likelihood of acceptance of HIV testing.

### Correlates of Maternal ARV Uptake

Factors associated with uptake of maternal ARVs are described in Table 3. Women who did not access any ARVs reported fewer ANC visits than those who used ARVs (median 2 vs. 4, p = 0.002), and they were less likely to have a partner who was tested for HIV (53% vs. 76% of those without vs. with ARV uptake). Among those who knew their partner's HIV status, having an HIV-positive partner was associated with uptake of maternal ARVs. Correlates of uptake of a complete course of maternal ARVs included more education (41% vs. 61% of those who had an incomplete vs. complete ARV course had finished primary school, p = 0.006). Women who accessed a skilled provider at delivery had increased uptake of ARVs specifically during the labor/delivery time point (p = 0.02). In multivariate analyses, adjusted for number of ANC visits, partner HIV testing, and access to a skilled provider at delivery, older age and a higher number of ANC visits during the last pregnancy were associated with uptake of any antiretrovirals for PMTCT. A full course of PMTCT was associated with completion of primary education. Adjusted prevalence ratios for each of the primary outcomes are detailed in Table 4.

**Table 3:** Reported uptake of maternal antiretrovirals among HIV-positive women.

*Uptake of Maternal Antiretrovirals*	*None*	*Any*		*No or Some*	*Full Course* *	
	*n(%) or med (IQR)*	*n(%) or med (IQR)*	*p-value*	*n(%) or med (IQR)*	*n(%) or med (IQR)*	*p-value*
	*39 (18.1)*	*177 (81.9)*		*99 (45.8%)*	*117 (54.2%)*	
**Demographic Information**						
Age	26 (23–33)	29 (25–32)	0.06	28 (25–32)	29 (25–32)	0.62
Marital status			0.71			0.86
Married monogamous	27 (69.2)	132 (74.6)		71 (71.7)	88 (75.2)	
Married polygamous	7 (18.0)	28 (15.8)		16 (16.2)	19 (16.2)	
Single	2 (5.1)	5 (2.8)		4 (4.0)	3 (2.6)	
Widowed	3 (7.7)	12 (6.8)		8 (8.1)	7 (6.0)	
Occupation			0.27			0.07
Unemployed	7 (18.0)	32 (18.3)		17 (17.4)	22 (19.0)	
Housewife	22 (56.4)	76 (43.4)		53 (54.1)	45 (38.8)	
Employed	10 (25.6)	67 (38.3)		28 (28.6)	49 (42.2)	
Completed primary education	16 (41.0)	96 (54.2)	0.16	41 (41.4)	71 (60.7)	0.006
Cattle ownership (≥1 in household)	19 (48.7)	70 (39.6)	0.37	40 (40.4)	49 (41.9)	0.89
Mobile phone (≥1 in household)	27 (69.2)	125 (70.6)	0.85	64 (64.7)	88 (75.2)	0.10
Roof type			0.72			0.89
Grass	23 (59.0)	97 (54.8)		56 (56.6)	64 (54.7)	
Corrugated iron sheet or better	16 (41.0)	80 (45.2)		43 (43.4)	53 (45.3)	
**Pregnancy Information**						
Number of times pregnant	4 (3–6)	4 (3–6)	0.33	4 (3–6)	4 (3–6)	0.66
ANC during last pregnancy	36 (92.3)	173 (97.7)	0.11	94 (95.0)	115 (98.3)	0.25
Timing of first ANC (months pregnant)	6 (5–7)	5 (4–6)	0.18	5 (4–6)	5 (4–6)	0.25
Number of ANC visits	3 (2–4)	4 (3–4)	0.002	3 (2–4)	4 (3–4)	0.23
**HIV Testing**						
First HIV test before learned was pregnant	11 (28.2)	79 (44.6)	0.07	37 (37.4)	53 (45.3)	0.27
Partner tested for HIV	18 (52.9)	117 (75.5)	0.01	59 (67.8)	76 (74.5)	0.33
Partner HIV-positive (among tested)	8 (44.4)	84 (75.7)	0.01	37 (63.8)	55 (77.5)	0.12
**Delivery Care**						
Skilled provider at delivery	13 (33.3)	88 (50.6)	0.08	40 (40.8)	61 (53.0)	0.10
**Knowledge and Beliefs**						
Is there any way to prevent HIV	31 (81.6)	161 (91.0)	0.14	80 (81.6)	112 (95.7)	0.001
Pregnant can women give HIV to baby	29 (74.4)	143 (81.3)	0.38	74 (75.5)	98 (83.8)	0.17
Can prevent MTCT of HIV	26 (66.7)	132 (75.4)	0.31	66 (67.4)	92 (79.3)	0.06

*Defined as uptake at antenatal, peripartum, and postpartum time points.

All women reporting HIV-positive status: including 43 from random sample and 173 from HBCT sample, Nyanza Province, Kenya (2011).

**Table 4:** Summary of Adjusted Prevalence Ratios (PR) for Accessing Steps of PMTCT Care.

	*ANC*	*HIV Testing*	*Maternal ARVs*	*Full Course Maternal ARVs*
	*PR (95%CI)*	*PR (95%CI)*	*PR (95%CI)*	*PR (95%CI)*
**Demographic Information**				
Age*			1.02 (1.00–1.03)†	
Occupation				
Unemployed		Ref		
Housewife		1.16 (1.00–1.35)		
Employed		2.73 (1.00–1.22)		
Completed primary education	1.09 (1.04–1.15)^¥^			1.37 (1.06–1.79)†
Cattle ownership (one or> in household)		1.08 (1.01–1.17)†		
Mobile phone ownership (one or> in household)		1.13 (1.00–1.26)†		1.19 (0.88–1.62)
Roof type				
Grass		Ref		
Corrugated iron sheet or better		1.12 (1.04–1.20)^‡^		
**Pregnancy Information**				
Number of times pregnant*	0.94 (0.89–0.99)†			
Number of ANC visits*			1.08 (1.03–1.13)^‡^	
**HIV Testing**				
Partner tested for HIV		1.13 (1.03–1.24)^‡^	1.16 (0.97–1.39)	
**Delivery Care**				
Skilled provider at delivery			1.10 (0.96–1.26)	1.16 (0.94–1.49)

Samples for each column described in previous tables; all variables in final adjusted models presented.

*Prevalence ratios for continuous and ordinal variables are for each one unit change in the variable.

† p<0.05, ^‡^p<0.01, ^¥^p<0.001.

### Correlates of Infant ARV

Administration of infant ARVs for PMTCT was highly correlated with maternal ARV uptake. While 160 of 174 (92%) women who took ARVs also administered them to their infant, 10 out of 39 (26%) women who did not take ARVs gave their infants ARVs (p<0.001).

### Sensitivity Analyses

Among women known to be HIV infected through previous home-based counseling and testing, 14 (5.7%) declined to answer questions about their HIV status and 60 (24.3%) reported to our field workers that they were HIV-negative. In a sensitivity analysis attempting to account for the low self-report among known HIV-positive women, in which we considered the most liberal (that all known HIV-positive women who did not self-report HIV-positive status *accessed* PMTCT services) and conservative (that all known HIV-positive women who did not self-report HIV-positive status *did not access* PMTCT) estimates, the expected range of any PMTCT ARV uptake was 61–87%, ARVs at all three time points was 35–66%, and infant ARV administration was 59–84%. In the HBCT sample, we further compared characteristics of women who reported or denied HIV-positive status in both univariate and multivariate models, and did not find differences in education, marital status, economic indicators, delivery provider, or HIV knowledge. HIV-positive women who did not report their status were younger than those who did (median 25.5 vs. 29 years) (Table 5).

**Table 5:** Sensitivity Analyses.

	*Admitted HIV Positive Status*	*Reported HIV Negative Status*		*Prevalence Ratio*
	*n (%) or med (IQR)*	*n (%) or med (IQR)*	*p-value*	*PR (95% Confidence Interval)*
	*n = 173*	*n = 60*		
Age (years)	29 (25–33)	25.5 (23–29.5)	0.003	
Age category (years)			0.01	0.53 (0.32–0.86)
15–20	7 (4.1)	9 (15.0)		
21–34	137 (79.2)	45 (75.0)		
35–46	29 (16.8)	6 (10.0)		
Marital status			0.24	
Married monogamous	128 (74.0)	44 (73.3)		
Married polygamous	28 (16.2)	9 (15.0)		
Single	7 (4.1)	6 (10.0)		
Widowed	10 (5.8)	1 (1.7)		
Occupation			0.59	
Unemployed	30 (17.5)	7 (11.9)		
Housewife	80 (46.8)	31 (52.5)		
Employed	61 (35.7)	21 (35.6)		
Completed primary education	95 (54.9)	30 (50.0)	0.55	
Skilled provider at delivery	71 (41.5)	23 (38.3)	0.76	
Is there any way to prevent HIV	155 (89.6)	51 (85.0)	0.35	
Pregnant can women give HIV to baby	138 (79.8)	47 (78.3)	0.85	
Can prevent MTCT of HIV	128 (74.0)	42 (71.2)	0.73	

Comparison of characteristics of women known to be HIV infected who disclosed or denied HIV status to field interviewers.

## Discussion

In this community-based study of women in western Kenya with a pregnancy during the prior calendar year, we observed high rates of accessing antenatal care at least once, with most reporting HIV testing offered at the first ANC visit. Using a community lens, our study suggests that facilities in this region serve almost all pregnant women at some point, and almost 90% of women with previously unknown HIV status reported receiving HIV testing during their pregnancy. Most (>80%) HIV infected women reported using ARVs for PMTCT. Because a large proportion of HIV infected women did not disclose their status to the field workers, complete ascertainment of ARV use was not possible. The denial of HIV status by some women to interviewers was unexpected and posed a challenge to complete ascertainment of ARV use in this community survey. It also suggests that acceptance of HIV testing in the home may not readily translate to subsequent disclosure of HIV status and access to HIV services. To account for this possibility, we presented the ranges of uptake in sensitivity analyses to compensate for women who did not self-disclose their HIV status. With the most conservative scenario assuming that none of these women accessed PMTCT, at least 60% of HIV-infected women would have received some pregnancy ARVs.

Although we noted excellent PMTCT coverage, there were several key opportunities for improvement. For example, entry into ANC, was often late, and uptake of a complete course of ARVs for PMTCT was low (54%). These numbers may be even lower considering the high number of known HIV-positive women who declined to reveal their status. This highlights challenges in engagement throughout the cascade, which are especially important since late uptake of ARVs for PMTCT is associated with a higher risk of transmission. [28] As maternal ARV uptake is correlated with infant ARV uptake, and skilled delivery is associated with maternal survival as well as uptake of ARVs, an emphasis on encouraging early uptake of ANC, access to skilled delivery, and ARVs for personal health, may dually promote maternal and infant well-being and survival.

Knowledge of HIV prevention and PMTCT were associated with uptake of HIV testing and maternal ARVs. Given the cross-sectional study design, it is impossible to know whether the knowledge or uptake came first. In our survey, only 52% of women reported that they thought MTCT of HIV could be prevented. Among HIV infected women, PMTCT knowledge was higher at 73%. Women who knew that ARVs could decrease MTCT were more likely to report having taken a complete ARV course than those who did not. This suggests that investing more in early counseling regarding PMTCT ARVs in clinics will be useful to support sustained adherence to ARVs. Community-based activities that stress the effectiveness of PMTCT in preventing new infections will be important to fully realize benefits of PMTCT.

Socioeconomic indicators correlated with uptake of interventions throughout the PMTCT cascade. Maternal education was associated with ANC attendance and maternal ARVs; and higher socioeconomic status also correlated with uptake of HIV testing and ARVs. These associations may reflect easier access to care among women with higher socioeconomic status, or a better understanding of the benefit of HIV testing or ARVs. Approximately 70% of women reported having mobile phone availability, the majority of which were shared within the household. Mobile phones are being assessed as a tool (*m*Health) to improve PMTCT. [29]–[31] Although assessed as a measure of socioeconomic status, our observation that women without mobile phones were less likely to have HIV testing or to take ARVs suggests that these women may need specialized attention. *mHealth* interventions, while a very promising approach to improving engagement in care, may need to supply phones to these higher risk mothers to facilitate uptake of PMTCT and other health services.

The association between awareness of partner HIV testing and uptake of maternal HIV testing and ARVs in our study is consistent with previous studies, which have noted associations between partner disclosure, [32] social support from a spouse, [33] partner attendance at antenatal HIV counseling and testing, [34] and uptake of or adherence to PMTCT. These studies have generated enthusiasm for partner involvement. However, it is possible that these strategies may inadvertently marginalize women without supportive partners, or increase risk for gender-based violence. For example, a requirement that male partners attend ANC in Uganda resulted in women paying men (often not their partners) to attend clinic with them. [35] Male partner involvement may be simply a proxy for maternal self-efficacy, partner support, and communication that fosters access to care. Further studies are needed to discern the impact of male partner interventions on PMTCT, including unintended consequences.

Consistent with other national surveys, fewer than half of the women in this survey accessed a skilled provider for assistance during delivery. [36] The delivery time-point also had the lowest level of ARV uptake. Efforts to improve access to skilled care during delivery may provide multiplicative benefit to maternal and neonatal morbidity and mortality, as well as PMTCT. Community-based efforts to enhance the ability of unskilled providers to refer and educate about PMTCT services could be considered in order to reach the large proportion of women who deliver outside of formal health facilities. [37] Movement towards Option B+, which provides lifelong therapy to all pregnant women, may further improve uptake at delivery.

This analysis had several strengths in that it utilized a community-based approach to assess uptake of health services. Regional home-based counseling and testing in the HDSS further allowed for increased sampling of HIV-positive women and verification of self-reported status.

Community based approaches, such as the existing HDSS household annual surveys, provide a tool to efficiently assess interventions received by women during their most recent pregnancy and sample women attending a variety of clinics or not accessing any clinical services. In demographic surveys that include routine HIV surveillance, it is possible to rapidly sample women who should have received PMTCT ARVs to understand whether women access PMTCT programs and systems are effectively providing PMTCT services.

In contrast to studies from Uganda and a multi-national study (Cameroon, Cote d'Ivoire, Zambia), [16] our community PMTCT coverage estimate did not appear to differ markedly from national facility-based estimates. [7] However, while able to access a broader sample of women than a clinic-based approach, our approach had limitations in using self-report in the absence of biological samples. In our case, many women who were recorded as HIV infected in the HBCT database did not report HIV-positive status to the interviewer in this home-based survey, despite recent acceptance of home-based testing and identification of interviewers as being from the same program. This suggests that either women did not want to reveal their status a new interviewer but may have actually accepted PMTCT, or that women may not have accepted PMTCT because of unwillingness to acknowledge or misunderstanding of their HIV-positive status at health facilities, similarly to during home interviews. Respecting the confidentiality of the participant and her right to not disclose to the interviewer was an important ethical consideration which would be present in any community-based survey. Despite these limitations, our coverage findings are remarkably similar to the 2012 Kenya AIDS Indicator Survey (KAIS) (87% uptake of HIV-testing; 72% antepartum prophylaxis; 69% peripartum prophylaxis; 75% postpartum prophylaxis; and 15% infant HIV infection).

Each of the possible approaches for assessing population-level uptake and health impact of HIV prevention services poses methodological challenges. Facility-based surveys may fail to assess outcomes of women who never access clinic-based services, while community-based approaches may fail to reach women who deny HIV status or decline HIV testing. Even with biological testing, 15% of respondents in the KAIS PMTCT surveys refused HIV testing. Furthermore, program indicators of uptake, while important in understanding issues related to engagement in care, are not equivalent to measures of program effectiveness. Findings from the PEARL Study demonstrated that program data in Cote D'Ivoire suggested markedly different rates of reported uptake of nevirapine (41%) compared to presence of nevirapine cord-blood samples collected from infants (16%). [38] Often this is a result of challenges in monitoring program data, but it also may reflect adherence challenges throughout the complete course of ARVs. Future larger-scale studies with biological markers of HIV status and antiretroviral coverage, and with approaches that better protect participant privacy such as computer-assisted self-interview tools, are indicated to more rigorously assess engagement in and coverage of PMTCT services.

## Conclusions

This household-based survey complements Kenyan facility-based assessments and observed similarly high PMTCT coverage estimates. However, although most HIV infected women received ARVs, fewer received a complete course, many started ANC late, and a surprising number of women declined to reveal their status. Involving partners or utilizing mobile phones may enhance PMTCT delivery, though care should be taken to avoid marginalization of women without supportive partners or access to mobile phone technology. Efforts specifically targeting stigma reduction around disclosure of HIV status and provision of ARVs during the labor and delivery period are necessary. Community driven strategies that encourage early uptake of ANC and skilled attendance at delivery, and that emphasize education about the effectiveness of PMTCT, may facilitate completion of ARVs and subsequent reductions in perinatal HIV transmission.
